# *Sacrificial Agent Gone Rogue*: Electron-Acceptor-Induced Degradation of CsPbBr_3_ Photocathodes

**DOI:** 10.1021/acsenergylett.1c02130

**Published:** 2021-12-27

**Authors:** Hye Won Jeong, Tamás Sándor Zsigmond, Gergely Ferenc Samu, Csaba Janáky

**Affiliations:** † Department of Physical Chemistry and Materials Science, Interdisciplinary Excellence Centre, University of Szeged, Aradi Square 1, Szeged H-6720, Hungary; ‡ ELI-ALPS, ELI-HU Non-Profit Ltd., Wolfgang Sandner street 3, Szeged H-6728, Hungary

## Abstract

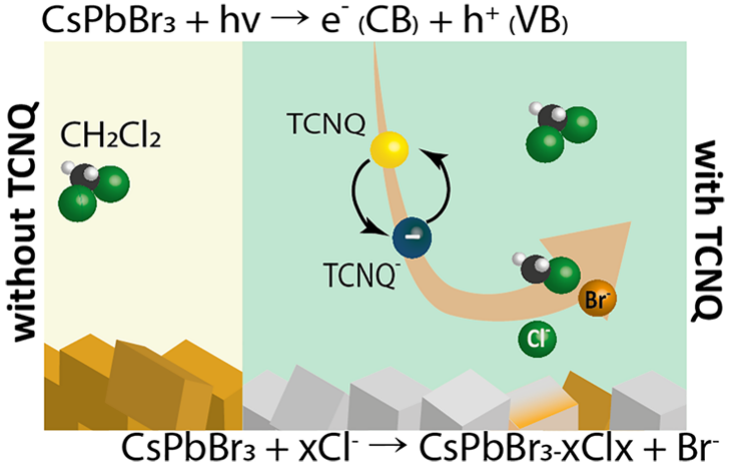

Lead halide perovskites (LHPs) have emerged as perspective materials for light harvesting, due to their tunable band gap and optoelectronic properties. Photocatalytic and photoelectrochemical (PEC) studies, employing LHP/liquid junctions, are evolving, where sacrificial reagents are often used. In this study, we found that a frequently applied electron scavenger (TCNQ) has dual roles: while it leads to rapid electron transfer from the electrode to TCNQ, enhancing the PEC performance, it also accelerates the decomposition of the CsPbBr_3_ photoelectrode. The instability of the films is caused by the TCNQ-mediated halide exchange between the dichloromethane solvent and the LHP film, during PEC operation. Charge transfer and halide exchange pathways were proposed on the basis of *in situ* spectroelectrochemical and *ex situ* surface characterization methods, also providing guidance on planning PEC experiments with such systems.

Lead-halide perovskites (denoted as LHPs) are promising materials for a multitude of technological applications, such as photovoltaics,^1−4^ light-emitting devices,^5,6^ and radiation sensing.^7,8^ These materials adopt the general formula of APbX_3,_ where the A site can be occupied by various cations (e.g., Cs^+^, MA^+^: CH_3_NH_3_^+^ or FA^+^: CH(NH_2_)_2_^+^) and the X site by halide anions (e.g., Cl^–^, Br^–^, or I^–^). Their remarkable properties can be mainly ascribed to their large extinction coefficient, long carrier diffusion length, and defect-tolerant crystal structure.^9,10^ The ease of preparing high-quality layers, together with their composition-tunable bandgap, makes these materials especially attractive.^5,11,12^ The extreme sensitivity of LHPs to various environmental factors (e.g., moisture, UV light, oxygen, and temperature), however, still inhibits their practical utilization. In the case of mixed compositions, light can also induce a phase segregation of halide ions, which is a unique phenomenon related to the LHP family.^13−15^ The instability of LHPs becomes even more pronounced in the case of photocatalytic (PC) and photoelectrochemical (PEC) solar energy conversion scenarios. In these cases, the presence of a solid/liquid interface greatly accelerates the degradation process. Several studies on LHP nanocrystals focused on the better understanding of the underlying mechanism of degradation, phase segregation, and ion exchange through this solid/liquid interface.^5,15−20^ LHPs are especially prone to halide exchange, which can even occur through the light-induced decomposition of the haloalkane-based electrolyte.^12,21,22^

PC solar energy harvesting using LHPs has been mainly studied in CO_2_ reduction in organic media and hydrogen evolution reaction in hydrohalic acids.^23−25^ Both PC and PEC applications, such as H_2_O splitting, CO_2_ reduction, and pollutant degradation, demand long-term operating times often under harsh conditions. The accumulation of charge carriers (especially holes) can induce halide motion within the LHP material and through the solid/liquid interface.^26,27^ Such carrier accumulation is also responsible for inducing photocorrosion.^28,29^ In PEC reactions, the use of sacrificial agents can prevent carrier accumulation on the electrode surface and often stabilize sensitive materials.^29^ This is the underlying reason these materials are often employed to assess the ultimate performance of photoelectrodes and reveal kinetic limitations in certain PEC reactions.^30,31^ This concept can be extended to redox mediators, which rapidly siphon the respective charge carrier from the electrode surface, and a potentially sluggish redox reaction takes place between the redox mediator and the substance afterward.^32^ The extreme sensitivity of LHPs toward water requires the use of organic solvents in electrochemical experiments. In such media, sacrificial agents such as methyl viologen, ferrocene, and *p*-benzoquinone have been widely employed to evaluate the characteristics of interfacial charge transfer.^33−36^ In this respect, liquid junction PEC cells with p-type MAPbI_3_ perovskites achieved a 6.1% optical to electrical energy conversion efficiency with *p*-benzoquinone as the scavenger species.^37^*In almost all PC and PEC studies, however, the fate of the redox-active molecule is neglected*. Notably, redox reactions can produce reactive intermediates or even products that can compromise the stability of LHP electrode materials.

In this paper, we present PEC experiments with the 7,7,8,8-tetracyanoquinodimethane (TCNQ) electron acceptor molecule in dichloromethane (DCM) medium with CsPbBr_3_ and MAPbI_3_ electrodes. *In situ* spectroelectrochemical measurements under continuous visible-light irradiation were carried out to evaluate the stability of the LHP photoelectrodes. During operation we observed a gradual Br^–^ to Cl^–^ anion exchange, which surprisingly was greatly accelerated in the presence of TCNQ. We monitored the compositional changes in both the solid and liquid phases. Finally, we uncovered the underlying mechanism of the anion exchange, including the exact role of TCNQ in these processes.

## Photoelectrochemical Behavior of CsPbBr_3_ Electrodes

**Figure 1:**
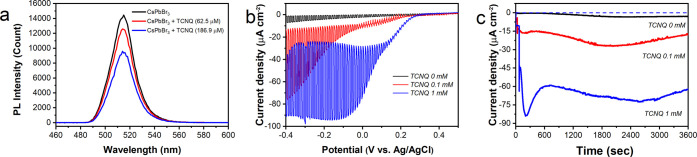
(a) Steady-state photoluminescence of CsPbBr_3_ NCs with and without added TCNQ upon 467 nm laser excitation. (b) Linear sweep photovoltammogram of FTO/CsPbBr_3_ electrodes with different concentrations of TCNQ, recorded with a 2 mV s^–1^ sweep rate and a light chopping frequency of 0.3 Hz. (c) Chronoamperometric current profiles recorded at −0.2 V vs Ag/AgCl. PEC measurements were performed in 0.1 M Bu_4_NPF_6_ DCM electrolyte using a spotlight illumination (λ > 400 nm) with 40 mW cm^–2^ intensity.

To confirm the electron-scavenging role of TNCQ, steady-state and time-resolved photoluminescence (PL) measurements were carried out on CsPbBr_3_ nanocubes (NCs) (Figure 1a and Figures S1 and S2). For the as-prepared NCs in DCM, the center of the PL peak was located at ∼520 nm. After the stepwise addition of the electron scavenger, a gradual quenching of the PL emission was observed, which reveals the prevalence of electron transfer from CsPbBr_3_ to TCNQ (Figure 1a). To reveal the effect of the added electron scavenger on the radiative decay of the excited state of CsPbBr_3_ NCs, time-resolved PL measurements were performed (Figure S1). As expected, the decay of the PL signal was accelerated in the presence of TCNQ (Table S1). We subjected the samples to prolonged PL measurements to rule out the possible degradation of CsPbBr_3_ NCs in the presence of light excitation and the electron scavenger species (Figure S2). Repeated measurement of the steady-state PL response and time-resolved PL traces prove that the differences observed between the with- and without-TCNQ cases are not caused by partial degradation of the CsPbBr_3_ NCs (Figure S2b).

PEC experiments of the CsPbBr_3_ electrodes were carried out in 0.1 M Bu_4_NPF_6_ DCM medium with and without added TCNQ. This medium is considered as an inert electrolyte for performing electrochemical experiments on LHPs.^37−40^ The cathodic photocurrent on the linear sweep photovoltammetry profiles signals the p-type behavior of CsPbBr_3_ electrodes (Figure 1b). When 0.1 mM TCNQ was added, a 5-fold increase in the photocurrent was observed (−0.2 V vs Ag/AgCl). When the electron scavenger concentration was increased to 1.0 mM, additional photocurrent enhancement was observed. At potentials more negative than 0.2 V vs Ag/AgCl, a cathodic dark current evolved in the presence of TCNQ.^41^ Chronoamperometric measurements were performed at −0.2 V vs Ag/AgCl (under spotlight illumination λ > 400 nm, 40 mW cm^–2^) for 1 h, to assess the stability of the observed photoresponse (Figure 1c). On these longer time scales the photocurrent was unstable, which signals that additional processes are occurring other than the PEC reduction of TCNQ. This was also apparent from the color change of the films, as the initially yellow layers turned white after the PEC measurements (Figure S3 inset).

On the UV–vis absorbance spectra of the layers, recorded before and after the chronoamperometric measurements, a shift to lower wavelengths of the absorption edge of CsPbBr_3_ was observed (Figure S3). This type of bandgap change is generally observed in connection with halide ion exchange, where the bromide content of the electrodes is gradually replaced by chloride (originating from the decomposition of DCM, as shown later).^22^ Several different factors can influence this halide exchange process (e.g., light, injected charge, reaction at the surface, etc.), and the fundamental chemistry that occurs at the CsPbBr_3_/electrolyte interface has to be understood.

## Tracking the TCNQ-Mediated Anion Exchange of CsPbBr_3_ Electrodes

Previously, we evaluated the electrochemical stability of LHP-based electrodes by performing *in situ* spectroelectrochemical measurements.^26,39^ Here we extended this technique by simultaneously irradiating the electrode surface with a spotlight, while performing the spectroelectrochemical measurements (Scheme S1). Both the decrease in the overall absorbance (dissolution) and the shift of the effective absorption edge from its initial value (compositional change) can be used as an indicator of stability. We performed stability tests in the electrolyte under four different conditions (Table S2): (i) only electrode immersion, (ii) light illumination, (iii) electrochemical polarization, and (iv) both light illumination and electrochemical polarization. This systematic approach allowed us to uncover the role of light illumination, injected charge, and surface reaction in the observed instability of CsPbBr_3_ photoelectrodes.

As the first step, we evaluated the chemical stability (CS) of CsPbBr_3_ electrodes immersed in the electrolyte in the presence of TCNQ for 30 min (Figure S4). The change in the effective absorption edge was negligible, which confirms that there is no significant influence of supporting electrolyte, solvent, or electron acceptor on the CS of the electrodes. As a next step, we assessed the photochemical stability (PCS) by illuminating the layers in the electrolyte (Figure S4). A 3 times larger shift in the effective absorption edge was observed in the presence of TCNQ, in comparison to the experiments without using the electron acceptor. In stark contrast, when an electrochemical stability (ECS) test was performed at −0.2 V vs Ag/AgCl, the addition of TCNQ suppressed the shift in the effective absorption edge of the CsPbBr_3_ electrodes. This peculiar behavior can be rationalized by considering the way charge carriers are generated under these two conditions. In the case of PCS tests, both electrons and holes are generated by light; however, when ECS tests are performed, only electrons are injected into CsPbBr_3_ electrodes. LHP materials show susceptibility to corrosion induced by both types of charge carriers.^29,39^ As shown by the PL measurement, TCNQ is able to scavenge electrons from the CB of CsPbBr_3_, which enhances the stability under electrochemical electron injection conditions.^42^ When PCS tests are performed, the same scavenging process leaves photogenerated holes in the VB of the material that might be more susceptible to an attack from chloride anions (from the decomposition of DCM).^43^

**Figure 2:**
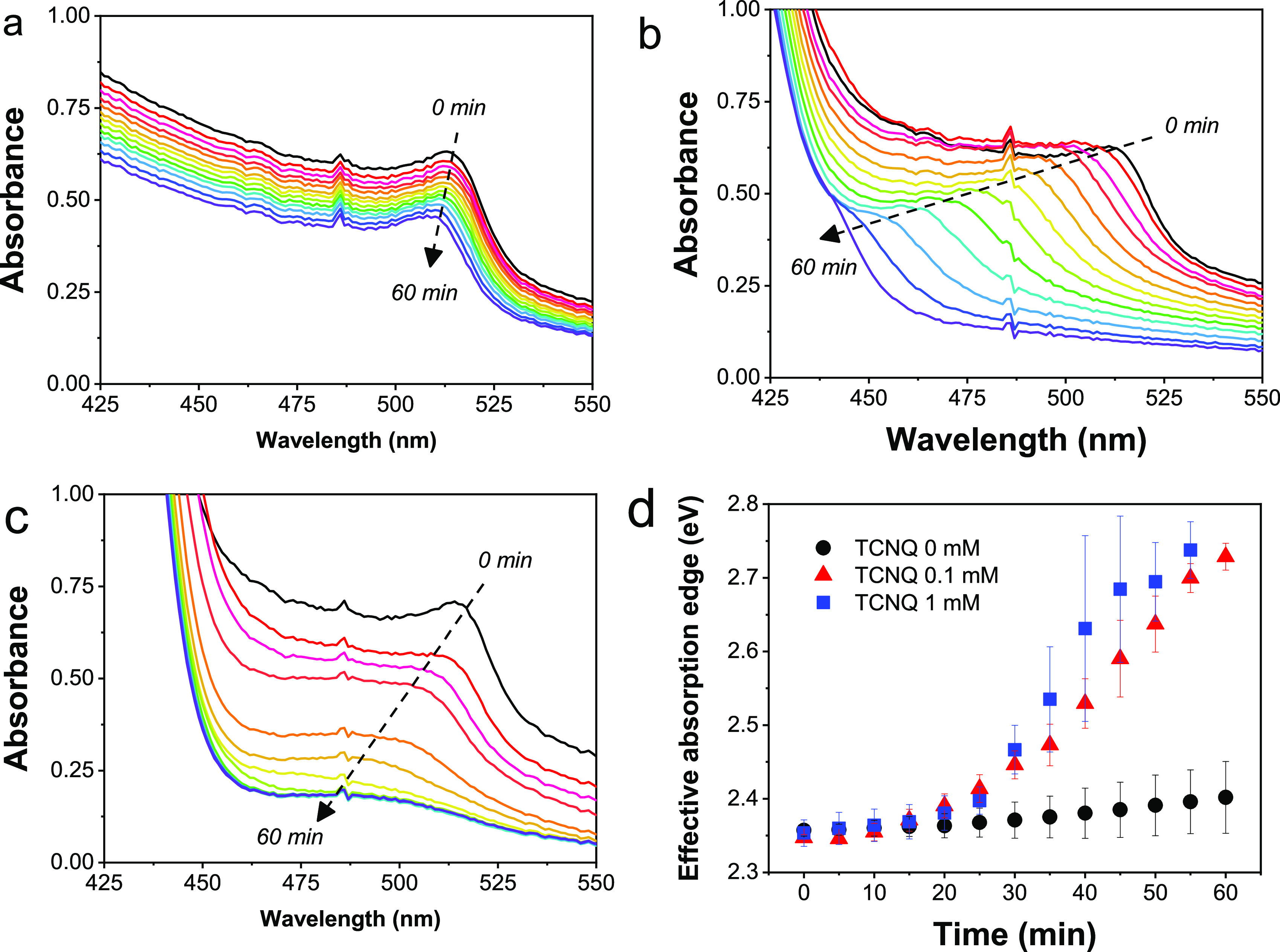
*In situ* UV–vis spectra of CsPbBr_3_ electrodes recorded during PEC operation in (a) 0.1 M Bu_4_NPF_6_ DCM electrolyte, containing (b) 0.1 mM and (c) 1 mM TCNQ as the electron scavenger. (d) Time evolution of the effective absorption edge of the CsPbBr_3_ film during PEC operation with an applied bias of −0.2 V vs Ag/AgCl under spotlight irradiation (40 mW cm^–2^) with different concentrations of TCNQ. Error bars represent the standard deviation of measurements on three different CsPbBr_3_ films.

In the case of PEC stability tests, the situation becomes more complex and convoluted, as light illumination generates both charge carriers (similarly to PCS tests), but the holes are simultaneously extracted under negative bias. The light absorption of the prepared CsPbBr_3_ films fall in the *A*_510 nm_ = 0.70 ± 0.13 range, summarized in Table S3 together with the recorded photocurrent. From this comparison, it is apparent that the recorded photocurrent is dominantly affected by the TCNQ concentration and not by the minor variations in the layer thickness. Figure 2 and Figure S5 show the *in situ* UV–vis absorption spectra of these CsPbBr_3_ photoelectrodes, during PEC experiments in electrolytes with and without TCNQ. Similarly to PCS tests, the effective absorption edge of CsPbBr_3_ electrodes shifted to shorter wavelengths, which was also accompanied by a slight decrease in the overall UV–vis absorption of the layer, which is the sign of dissolution. To follow the extent of chloride incorporation into the CsPbBr_3_ films, the evolution of the effective absorption edge was monitored (Figure 2d). A brief description of the determination method is shown in Figure S6 in the Supporting Information. Even without added TCNQ, a minor effective absorption edge shift (∼0.05 eV in 1 h) was observed for the CsPbBr_3_ films (Figure 2d). When these experiments were performed on a longer time scale, the absorption edge shifted from 540 to 450 nm, signaling the complete exchange of bromide to chloride (Figure S5). The addition of TCNQ greatly accelerates this halide exchange process (Figure 2b,d). This shows that the employed electron scavenger is not an innocent bystander in these processes and plays an important role in accelerating the chloride incorporation. When the concentration of TCNQ was increased, the rate of the effective absorption edge shift remained identical, but the absorption of the film disappeared after 40 min (Figure 2c). The thickness of the CsPbBr_3_ electrode can have an influence on the rate of this halide exchange process. We carried out thickness-dependent PEC measurements in the presence of TCNQ (Figure S7). These measurements revealed that in the case of thick CsPbBr_3_ films (562 nm estimated layer thickness) only partial halide exchange occurs, as the process becomes surface-limited. Throughout the paper we present results on CsPbBr_3_ films having *A*_510 nm_ = 0.70 ± 0.13, which corresponds to an ∼260 nm average layer thickness, to ensure that complete halide exchange can be realized.

**Figure 3:**
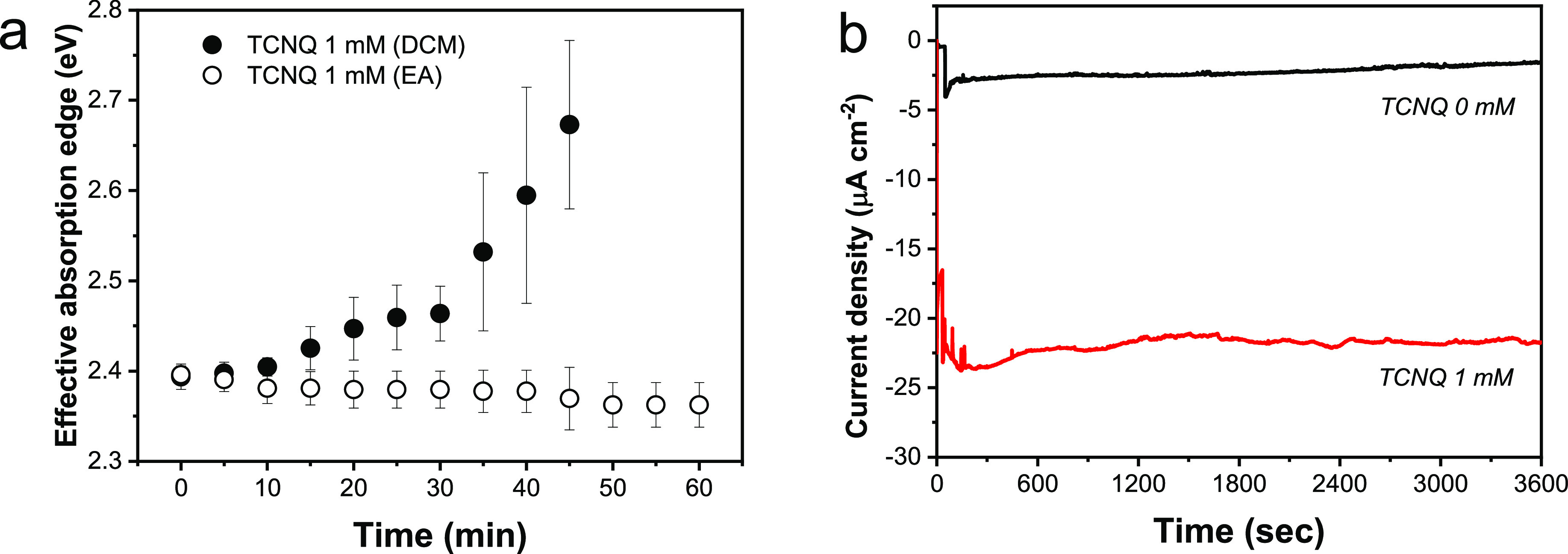
(a) Time evolution of the effective absorption edge of CsPbBr_3_ films during PEC operation with an applied bias of −0.2 V vs Ag/AgCl under spotlight irradiation (40 mW cm^–2^) with 1 mM TCNQ in DCM (0.1 M Bu_4_NPF_6_) and EA (0.01 M Bu_4_NPF_6_) electrolytes. Error bars represent the standard deviation of measurements on three different CsPbBr_3_ films. (b) Time-profiled photocurrent collected during PEC operation in EA medium.

To confirm that the decomposition of DCM is the source of chloride ions, we changed the solvent to ethyl acetate (EA). Figure 3a compares the variation of the effective absorption edge during PEC measurements in EA- and DCM-based electrolytes. In this chloride-free electrolyte, there was no effective absorption edge change for 1 h. The CsPbBr_3_ electrodes retained their photoresponse in this medium, as shown by chronoamperometric measurements under illumination (Figure 3b). These measurements also reveal that TCNQ can act as an electron acceptor in this medium as well, and there is no direct interaction between the reduced form of TCNQ and the perovskite film.

## Compositional Changes of CsPbBr_3_ Electrodes during Halide Exchange

**Figure 4:**
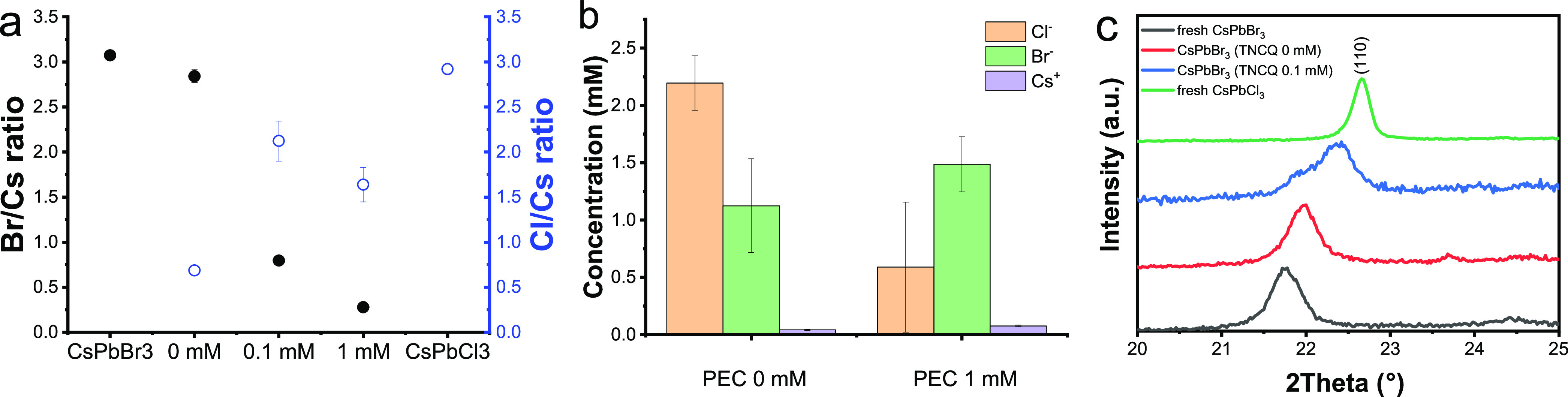
(a) X/Cs (X = Br, Cl) ratio of CsPbBr_3_ films derived from elemental analysis from EDS after PEC operation at −0.2 V vs Ag/AgCl after 1 h in electrolytes containing different amounts of TCNQ. As a comparison, the pristine CsPbX_3_ films is also displayed. (b) Quantification of Cs^+^, Cl^–^, and Br^–^ ions by ion chromatography in the solution phase collected after the PEC test. In both cases the error bars represent the standard deviation of three individual measurements. (c) X-ray diffraction patterns in the range 2θ = 20–25° of pristine CsPbBr_3_ and CsPbCl_3_ films together with CsPbBr_3_ photoelectrodes after 1 h of PEC operation in 0.1 M Bu_4_NPF_6_/DCM medium with and without TCNQ with an applied bias of −0.2 V vs Ag/AgCl under spotlight (λ > 400 nm) illumination with 40 mW cm^–2^.

To correlate the composition of CsPbBr_3_ films with the changes observed in the UV–vis spectra, we carried out *ex situ* XRD, XPS, and EDS measurements after PEC studies. Simultaneously, we examined the electrolyte composition using ion chromatography (IC) and quantified the Cs^+^, Br^–^, and Cl^–^ contents of the liquid phase. Figure 4a shows the halide to cesium ratio of the films determined by EDS, after a 1 h PEC measurement. In the pristine CsPbBr_3_ and CsPbCl_3_ films, the Br/Cs and Cl/Cs ratios were ∼3.0, as expected. After the PEC measurements, a decrease in the Br/Cs ratio (and increase in Cl/Cs ratio) was observed in all cases, which mirrored the effective absorption edge changes extracted from the UV–vis spectra.

We explored the solution-phase composition by performing IC after PEC measurements (Figure 4b). An increased amount of dissolved Cs^+^ was found after PEC measurements in 1 mM TCNQ-containing medium in comparison to its electron-scavenger-free counterpart. This shows that the dissolution rate was much higher in the presence of TCNQ, which can be responsible for the overall absorbance loss (Figure 2c). Note that some dissolved Cs^+^ was also present after a 1 h PEC operation in the electrolyte without any added TCNQ. We also monitored the concentration of Br^–^ and Cl^–^ in the solution phase. In the presence of TCNQ we observed an increased amount of Br^–^ in the electrolyte, which signals Br^–^ expulsion from CsPbBr_3_ films. Interestingly, the Cl^–^ concentration in the electrolyte shows an opposite behavior. This might signal that the generated Cl^–^ is gradually taken up by the CsPbBr_3_ film to form mixed compositions (CsPbBr_3–*x*_Cl_*x*_) in the presence of TCNQ. Note that the Cs^+^ content of the solution phase was significantly smaller than those of any of the halide ion concentrations. This shows that anion exchange is more dominant than dissolution of the film.

The halide exchange was also confirmed by X-ray diffraction measurements (Figure 4c and Figure S8) on CsPbBr_3_, CsPbCl_3_, and used samples after PEC operation without and with 0.1 mM TCNQ-containing Bu_4_NPF_6_/DCM solution. All CsPbBr_3_ perovskite related peaks shifted to higher 2θ angles after PEC measurements, which signals compression of the perovskite lattice (see the magnified region of the (110) peak in Figure 4c) caused by the chloride incorporation. The halide exchange was more pronounced in the presence of TCNQ, confirming that TCNQ accelerates the halide exchange process. These findings are in good agreement with the EDS results, and therefore we can conclude that the halide exchange is not confined to the surface of these thin layers.

**Figure 5:**
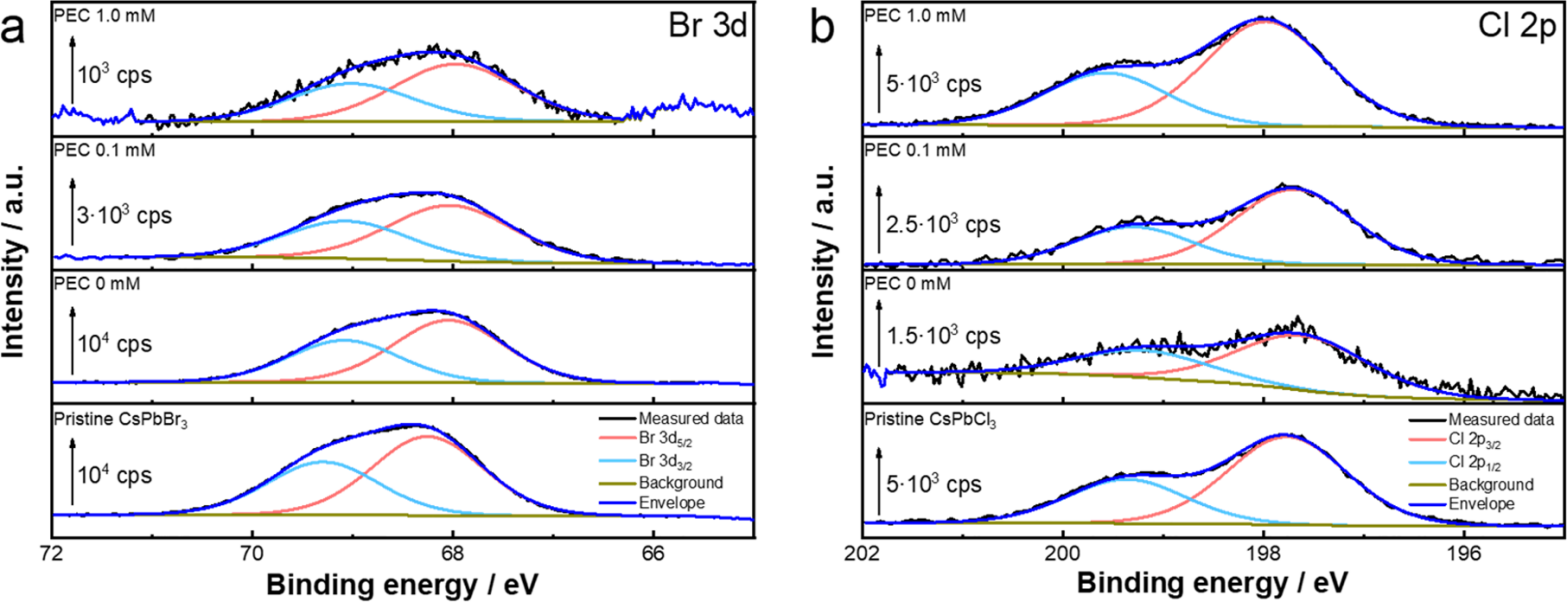
High-resolution XPS spectra of the (a) Br 3d and (b) Cl 2p regions of the CsPbBr_3_ electrodes after PEC measurements. The arrows indicate the intensity of each recorded spectrum.

To gain further insights into the halide-exchange-driven degradation process, the surface composition of the films was analyzed after the PEC measurements, via *ex situ* X-ray photoelectron spectroscopy (XPS) measurements (Figure 5 and Figure S9). The high-resolution scans of the bromide 3d region (Figure 5a) revealed a decreasing bromide content (3d_5/2_ at 68.26 eV) of the films in comparison to pristine CsPbBr_3_ films (Table S4). In parallel, the chloride 2p region (Figure 5b) shows an increase in the chloride content (2p_3/2_ at 197.76 eV) of the films. As the TCNQ concentration was increased, gradually more chloride was incorporated into the perovskite lattice (Table S4). Interestingly, XPS also reveals the presence of surface fluoride on the sample surface after the PEC measurements. A closer inspection of the F 1s region (Figure S9a) shows the presence of metal–fluoride bonds (683.4 eV). Simultaneously, a broadening of the Pb 4f core level (Figure S9b) is visible, where an additional Pb component (at 138.9 eV) emerges. This signals the formation of PbF_2_ on the sample surface, and this process also becomes more prevalent in the presence of TCNQ. This indicates that not only DCM but also the PF_6_^–^ from the conducting salt is decomposed during PEC operation. A similar instability of the PF_6_^–^ anion was shown by both radiolysis and electrochemical experiments in Li^+^ batteries.^44,45^ To prove this degradation pathway, we exchanged the conducting salt to 0.01 M Bu_4_NBPh_4_ (Figure S10). As expected, these measurements show the absence of fluoride on the sample surface (Table S5). However, the bromide–chloride anion exchange is still visible, revealing that the decomposition pathway leading to PbF_2_ formation is not initiating the halide exchange. SEM images recorded for the films also show the restructuring of the surface of these samples after PEC measurements in the presence of TCNQ (Figure S11). Voids and different-shaped crystals can be observed after halide exchange.

## Solution Species Involved in Halide Exchange

All previous measurements point toward the reduced form of TCNQ being an active participant in both accelerating bromide–chloride exchange of CsPbBr_3_ films and inducing decomposition of the conducting salt. To gain information on the fate of TCNQ during the PEC test, we recorded UV–vis absorption spectra of both DCM- and EA-based solutions (Figure S12), using a 90° rotated electrochemical cell (Scheme S1b). It was possible to distinguish among the different forms of TCNQ^*n*–^ (*n* = 0, 1, 2) in the recorded UV–vis spectra.^33,46^ Briefly, TCNQ absorbs light above 400 nm and TCNQ^–^ has two absorption peaks at 750 and 850 nm, while the absorption peak of TCNQ^2–^ (or F-TCNQ^2–^) is located at 486 nm. Without TCNQ, there was no change in the UV–vis spectra of the solution phase (Figure S12a,b). There was also no sign of any detached/dissolved CsPbBr_3_ or any expelled species. With the addition of 0.1 mM TCNQ, a markedly different behavior was observed (Figure S12c,d). In the case of DCM medium (Figure S12c), there was no sign of TCNQ^2–^ (highly reduced species). In stark contrast, in EA medium a steady increase of this form was observed (Figure S12d). This signals that in EA multiple electron transfer steps can occur from CsPbBr_3_ electrodes to TCNQ, as no other reaction consumes TCNQ^–^. In the case of DCM, however, this reduced form is consumed in the reaction with the DCM solvent, producing chloride anion.^43^

## Proposed Mechanism of Halide Exchange

**Scheme 1:**
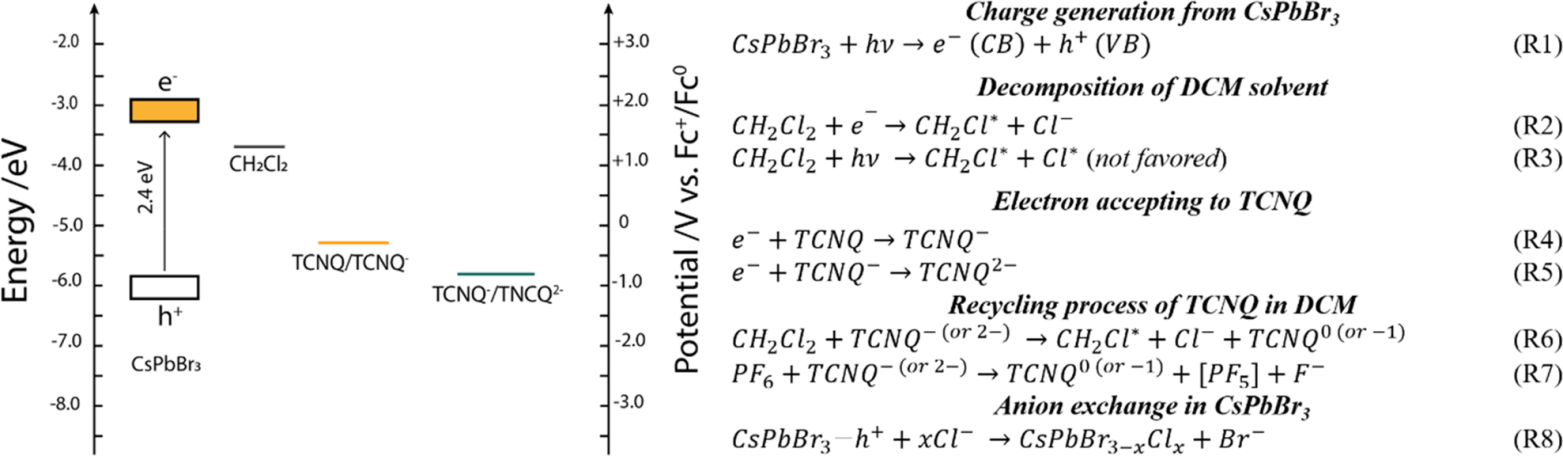
Energy Level Diagram of CsPbBr_3_ and Reduction Potential of Halogenated Solvent^22^ and TCNQ Species^4^

On the basis of the previous observations, we propose a possible mechanism of bromide–chloride exchange in CsPbBr_3_ with DCM in the presence of the electron acceptor TCNQ (Scheme 1). After light irradiation, electrons and holes are generated in CsPbBr_3_ films. The illumination used is also capable of decomposing a small portion of DCM directly (λ ≤ 350 nm),^47^ forming chloride anions (Scheme 1, eq R3). Therefore, there is a slow anion exchange even without the addition of TNCQ in the DCM-based solution (as seen in the PC and PEC cases). In contrast, when TCNQ is present in DCM, the photogenerated electrons are transferred from CsPbBr_3_ to the electron scavenger, producing reduced TCNQ species (TCNQ^–^ and TCNQ^2–^ in Scheme 1, eqs R4 and R5). In a subsequent step, the TCNQ^–^ can react with DCM, decomposing it and ultimately increasing the concentration of chloride anion near the electrode surface (Scheme 1, eq R6). This causes the accelerated anion exchange process of CsPbBr_3_ film in the presence of TCNQ. Importantly, the localization of a hole is also necessary to induce the chloride incorporation. This notion was further verified with an experiment, where a hole-transport layer (i.e., CuI) was inserted between the FTO support and the CsPbBr_3_ film, and the effective band edge shift disappeared (Figure S13). In addition, when only electrons were injected into the CsPbBr_3_ films, no halide exchange was observed (see electrochemical stability tests in Figure S4). TCNQ^–^ was also found to be responsible for decomposing the PF_6_^–^ anion used as the conducting salt (Scheme 1, eq R7). Through these reactions the formation of fluoride species can also influence the instability of LHP photoelectrodes.

In conclusion, we evaluated the PEC performance of CsPbBr_3_ films in the presence of the electron scavenger TCNQ in DCM. We encountered the instability of the films, which resulted in a drastic color change of the electrodes. We linked this alteration to chloride incorporation into the CsPbBr_3_ lattice, forming mixed halide compositions (CsPbBr_3–*x*_Cl_*x*_). The compositions after the halide exchange reaction determined by different material characterization techniques show good correlation with each other (Table S6). The decomposition of the employed DCM solvent was responsible for releasing chloride into the electrolyte, and this process was accelerated by the presence of the reduced form of TCNQ. This observation revealed the dual nature of the electron scavenger: beyond siphoning the electrons from CsPbBr_3_, it accelerated the halide exchange process through secondary reaction pathways. We extended our study to MAPbI_3_ photoelectrodes (Figures S14 and S15 and discussion therein) and revealed similar instability in the presence of TCNQ. These findings have a broader implication in PEC reactions using various scavenger species. After the electron transfer step from the photoelectrode to the sacrificial reagents, the fate of these molecules is often neglected. Through the formation of reactive species, they can participate in different reactions and ultimately compromise the stability of sensitive photoelectrodes, such as LHPs. This behavior of redox couples is complex and controversial, as instead of suppressing photocorrosion, an opposite effect can be achieved.

## Supporting Information Available

Experimental methods and additional spectroelectrochemical, XPS, and SEM data (PDF)
