# Supplementary material for: Sacrificial Agent Gone Rogue: Electron-Acceptor-Induced Degradation of CsPbBr3 Photocathodes
Source: ACS Energy Lett. 2021 Dec 27;7(1):417–24. doi: 10.1021/acsenergylett.1c02130 (PMC8762702; doi:10.1021/acsenergylett.1c02130)
Supplement: Supplementary file 1 — nz1c02130_si_001.pdf [file nz1c02130_si_001.pdf]

Supporting Information

Sacrificial Agent Gone Rogue – Electron Acceptor Induced Degradation of $CsPbBr_3$ Photocathodes

Hye Won Jeong[1,*], Tamás Sándor Zsigmond[1], Gergely Ferenc Samu[1,2], Csaba Janáky[1,2,*]

[1]Department of Physical Chemistry and Materials Science, Interdisciplinary Excellence Centre, University of Szeged, Aradi Square 1, Szeged, H-6720, Hungary

[2]ELI-ALPS, ELI-HU Non-Profit Ltd., Wolfgang Sandner street 3., Szeged, H-6728, Hungary

*Address correspondence to these authors: janaky@chem.u-szeged.hu and hwj1012@gmail.com Twitter: @JanakyLab

Chemicals and materials

$CsPbX_3$ nanocubes (NCs) were synthesized using cesium carbonate (Cs_2CO_3, 99.9%, metals basis), lead (II) bromide ($PbBr_2$, Alfa Aesar, 99.999%), lead (II) chloride ($PbCl_2$, Sigma Aldrich, powder, 98%), oleic acid (OlAc, Sigma Aldrich, technical grade, 90%), oleylamine (OlAm, Sigma Aldrich, technical grade, 70%), 1-octadecene (ODE, Sigma Aldrich, 90%), hexane (Sigma Aldrich, 95%), 2-propanol (VWR, ≥99.7%).

$MAPbI_3$ was prepared by using lead (II)-iodide (PbI_2, Sigma-Aldrich, Ultradry, beads, −10 mesh, 99.999% trace metals basis), methylammonium-iodide (MAI, Dyesol) dimethylformamide (DMF, Sigma-Aldrich, anhydrous, 99.8%), diethyl-ether (DEE, Sigma-Aldrich, for HPLC, ≥99.9%, inhibitor-free).

For photoelectrochemical experiments, dichloromethane (DCM, Sigma Aldrich, anhydrous, >99.8%, contains 40-150 ppm amylene as stabilizer) and ethyl acetate (EA, VWR, ≥ 99%) were used with tetra-n-butylammonium hexafluorophosphate (Bu_4NPF_6, Alfa Aesar, 98%) or tetra-n-butylammonium tetraphenylene borate (Bu_4NBPh_4, 99%) as the supporting electrolyte. DCM and EA were dried with 3Å molecular sieves (Sigma Aldrich) overnight and Bu_4NPF_6 was dried in a vacuum oven at 180 ℃ for 6 hours. 7,7,8,8-tetracyanoquiondimethane (TCNQ, Sigma Aldrich, 98%) was used as an electron acceptor, dried at 150 ℃ for 6 hours under vacuum. It is important to use “fresh” TCNQ for the experiments. When TCNQ is stored for a long period, yellowish particles might exist in it. Such non-fresh sample shows a strong response between 700 nm and 900 nm which is assigned to $TCNQ^-$.

Preparation of $CsPbX_3$ NCs and films

$CsPbBr_3$ NCs were synthesized via hot-injection method [1]. For the Cs-oleate precursor, Cs_2CO_3 (0.1628 g) was dissolved in a mixture of ODE (8 ml) and OlAc (0.5 ml) in a 50 ml 3-neck round bottom flask. The solution was degassed under vacuum for 1 hour at 120 °C and further increase to 140 °C was done under a N_2 atmosphere. For the lead-bromide precursor, $PbBr_2$ (0.345 g) and ODE (25 ml) were mixed in a separate 3-neck round bottom flask, and similarly degassed under vacuum for 1 hour at 120 °C. OlAc (2.5 ml) and OlAm (2.5 ml) were added to the solution under N_2 atmosphere and a clear solution was obtained. The temperature was raised to 170 °C, and the as-prepared Cs-oleate precursor (2 ml) was swiftly injected into the Pb-precursor containing flask and immediately cooled in a water-ice bath within 5 sec. When the temperature reached ~25 °C, the crude solution was centrifuged at 10 000 rpm for 10 min. The precipitate were re-dispersed in hexane and centrifuged at 3000 rpm. For the layer preparation the supernatant was collected. $CsPbCl_3$ NCs were synthesized using the same procedure, just replacing $PbBr_2$ with $PbCl_2$ (0.261 g).

For the preparation of $CsPbBr_3$ films, fluorine-doped tin oxide (FTO) glass was used as the substrate. As a cleaning step the FTO slides were ultrasonicated in ethanol, 2-propanol and deionized water for 10 min each. The $CsPbX_3$ NC stock solution was mixed with 2-propanol (vol. ratio = 1:2) and the mixed solution was centrifuged at 10 000 rpm for 10 min. The collected precipitate was re-dispersed in hexane with 16.6 vol% of 2-propanol. The spin coating procedure was 1500 rpm for 30 sec, where 35 μl of the $CsPbX_3$ solution was used. Dropping the $CsPbX_3$ solution was repeated 5 times and the film were heat-treated at 100 °C for 5 min. This whole procedure was repeated until a layer absorbance of $A_{510\ nm} = 1.0$ was reached (average of 30 cycles). For the CuI/$CsPbBr_3$ films, the preparation method of CuI hole transport layer was reported in our previous work [2].

Preparation of $MAPbI_3$ films

The $MAPbI_3$ perovskite layers were prepared by a one-step method, where all the precursors were dissolved in DMF, and subsequently spin-coated on the FTO substrates. The spin-coating solution consisted of 1.5 M PbI_2 and MAI for PEC measurements and 0.38 M PbI_2 and MAI for spectroelectrochemical measurements. Prior to spin-coating the solution was left to stir for 1 h at room temperature and was filtered with an inorganic membrane filter (0.2 μm pore size, G8549141, Whatman) before use. During spin-coating, the samples were subjected to an antisolvent treatment step with DEE. After the spin-coating the samples were immediately transferred to a hot-plate preheated at T=65 °C for 1 min to ensure evaporation of the antisolvent. This was followed by an annealing step at T=100 °C for 2 min.

Characterization of $CsPbX_3$ films

The composition and morphology of the as-prepared $CsPbBr_3$ film was carried out using field-emission scanning electron microscopy (FE-SEM, Hitachi S-4700) and energy-dispersive X-ray spectroscopy (EDS, RONTEC). Steady-state photoluminescence and time-resolved photoluminescence (PL) decay were measured using Horiba DeltaPro with a 467 nm laser source and photoluminescence decay was monitored at 518 nm. To quantify the effect of the electron scavenger on the charge carrier lifetime, we performed kinetic analysis of the decay curves. A biexponential decay was used to describe the observed decay traces, and the results are summarized in (**Table S1**). The dissolved Br^- and Cl^- concentration was determined using ion chromatography (Shimadzu Prominence LC-20ad, Shodex 5U-YS-50 column for cation, and NI-424 5U for anion detection). The mobile phase for cation- and anion-detection was 4 mM methanesulfonic acid (1.0 ml min^{-1}) and 2.5 mM phthalic acid + 2.3 mM aminomethane respectively, with an eluent flow rate of (1.0 ml min^{-1}).

X-ray diffraction (XRD) patterns were measured using Rigaku Miniflex II instrument, operating with a Cu Kα, radiation source (λ = 0.1541 nm). X-ray photoelectron spectroscopy (XPS) was performed with a SPECS instrument equipped with a PHOIBOS 150 MCD 9 hemispherical analyzer. The analyzer was in FAT mode with 20 eV pass energy. The Al Kα radiation (hν = 1486.6 eV) of a dual anode X-ray gun was used as an excitation source and operated at 150 W power. Ten scans were averaged to get a single high-resolution spectrum. The adventitious carbon peak was set at 284.8 eV in all cases. For spectrum evaluation, CasaXPS commercial software package was used.

Photoelectrochemical and spectroelectrochemical measurements

Photoelectrochemical measurements were carried out using three-electrode system in a home-made spectroelectrochemical cell in 0.1 M Bu_4NPF_6 DCM solution with a Biologic VMP-300 potentiostat/galvanostat. The $CsPbBr_3$ electrodes functioned as the working electrode, a Pt wire as the counter electrode, and a Ag/AgCl wire as a pseudo-reference electrode. The Ag/AgCl pseudo-reference was calibrated by measuring the redox potential of ferrocene (1 mM) in 0.1 M Bu_4NPF_6 DCM and a half-wave potential of ~ 0.49 V ± 0.01 was determined. Linear sweep photovoltammograms were performed in 0.1 M 0.1 M Bu_4NPF_6 DCM electrolyte using spot-light source (Fiber-Lite A3000, 40 $mWcm^{-2}$). A scan rate of 2 $mVcm^{-1}$ was used under chopped illumination with a frequency of 0.3 Hz. Spectroelectrochemical measurements were performed under simultaneous spot-light source illumination with a UV-vis spectrophotometer (Agilent, HP 8453). The UV-vis spectra were collected every 2 sec without closing the shutter during measurement. The change of $CsPbBr_3$ film during test was estimated from direct optical band gap of $CsPbBr_3$ film converted using the Tauc method [3]. An example for this method and a brief description cis shown later (**Figure S6**).

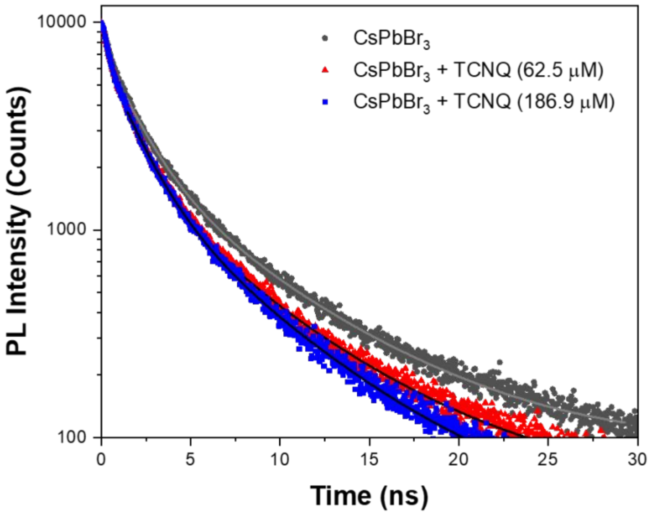

Figure S1. Time-resolved photoluminescence decay of 0.03 μM $CsPbBr_3$ NCs in DCM/hexane (20:80 vol.%) media excited at 467 nm. Time-resolved photoluminescence decay was collected at 518 nm emission.

The PL decay was fitted with two components corresponding to an interactive (short lifetime) and non-interactive (long lifetime) states, respectively.[4]

Table S1. Fitting parameters of the photoluminescence decay curves for $CsPbBr_3$ NCs with and without TCNQ in DCM/hexane (20:80 vol.%) media.

Sample	t1 (ns)	t2 (ns)	b1 (%)	b2 (%)	Average lifetime (ns)
$CsPbBr_3$	1.776	6.673	43.22	56.78	3.05
$CsPbBr_3$ + TNCQ (62.5 μM)	1.654	6.274	47.07	52.93	2.71
$CsPbBr_3$ + TNCQ (186.9 μM)	1.662	5.908	48.21	51.79	2.65

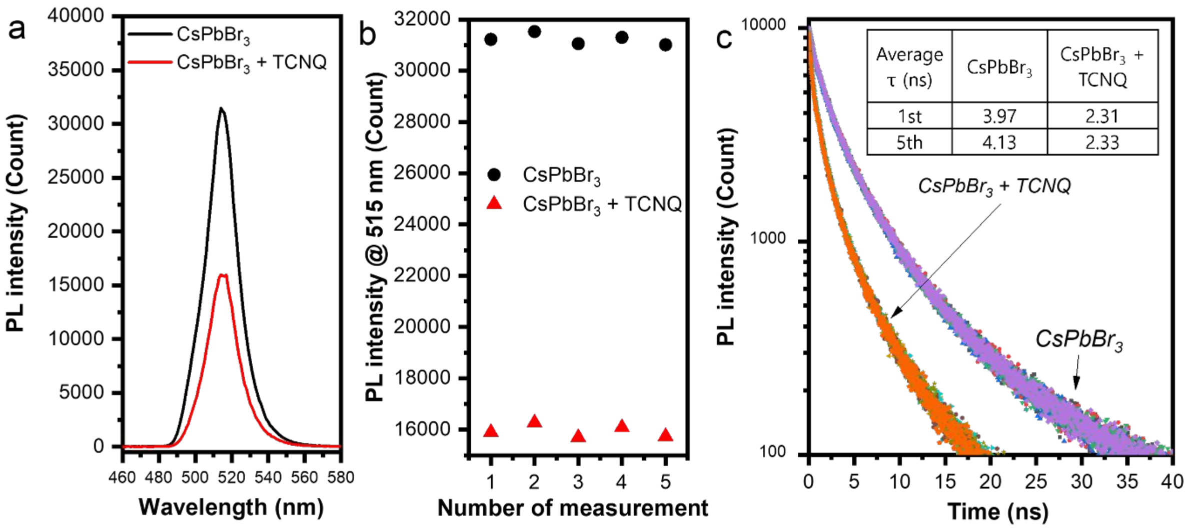

Figure S2. (a) Steady-state photoluminescence spectra (b) PL peak intensities recorded after five consecutive measurements at 515 nm, (c) and time-resolved photoluminescence decay curves recorded for 0.03 μM $CsPbBr_3$ NCs in DCM/hexane (20:80 vol.%) media with and without added 200 μM TCNQ excited at 467 nm. The time-resolved PL decay traces were collected at 508 nm emission. Inset table shows the average lifetime (determined from a biexponential fit) of 1st and 5th trials.

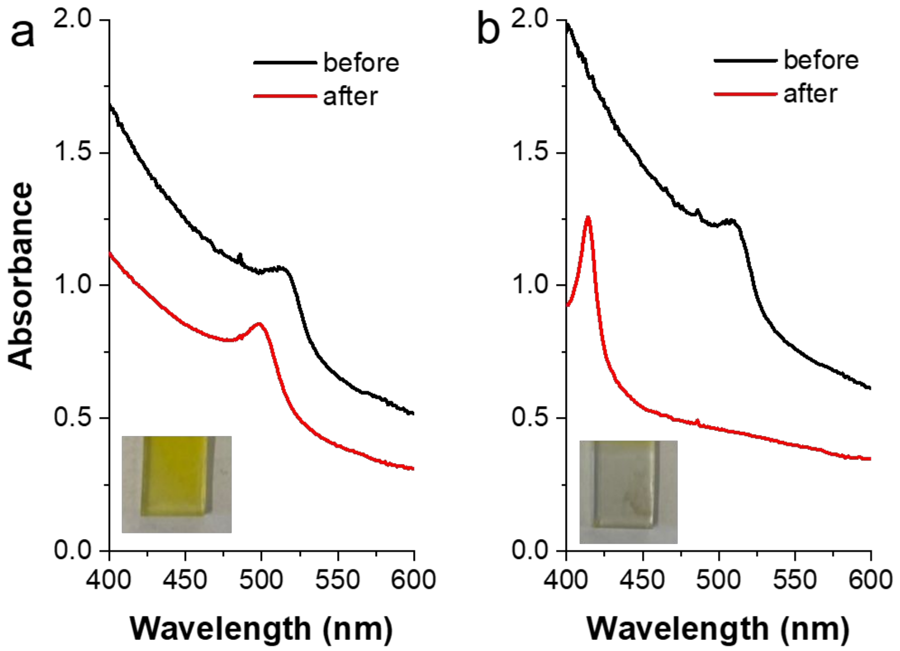

Figure S3. UV-vis absorption spectra collected before and after applying -0.2 V vs. Ag/AgCl for 60 minutes in 0.1 M Bu_4NPF_6 containing DCM media A: without and B: with added 1mM TCNQ. Inset: photographs of the $CsPbBr_3$ films after measurement.

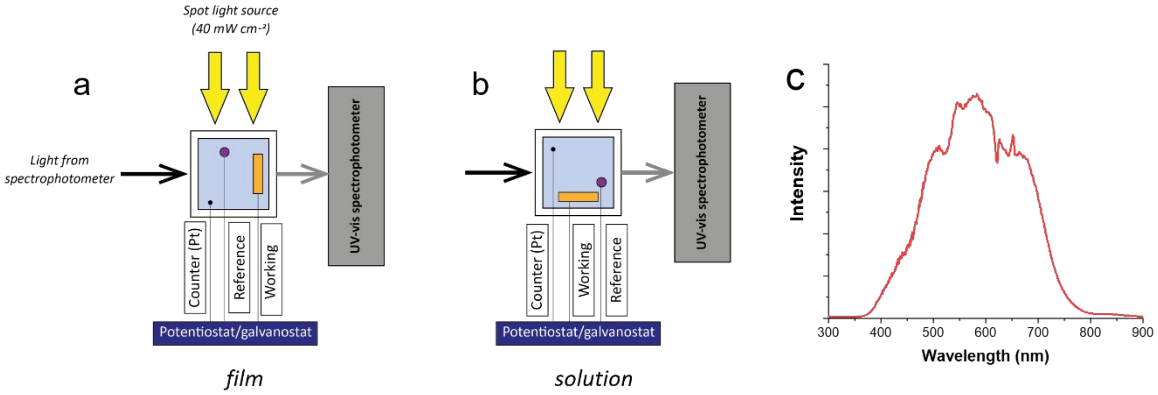

Scheme S1. Schematic representation of the set-up used for in-situ UV-vis absorption measurements of (a) film and (b) electrolyte with simultaneous light irradiation. (c) spectra of the spot-light source.

Table S2. Experimental details and abbreviation of measurement.

	Experimental details
Chemical stability test (CS)	The film was immersed in the electrolyte for 30 min.
Photochemical stability test (PCS)	The film was immersed in the electrolyte and irradiated with a spot-light source (40 mW cm^{-2}) for 30 min.
Electrochemical stability test (ECS)	The film was immersed in the electrolyte and -0.2 V vs. Ag/AgCl electrical bias was applied for 30 min.
Photoelectrochemical stability test (PECS)	The film was immersed in the electrolyte and -0.2 V vs. Ag/AgCl electrical bias was applied, while illuminated by a spot-light source (40 mW cm^{-2}).

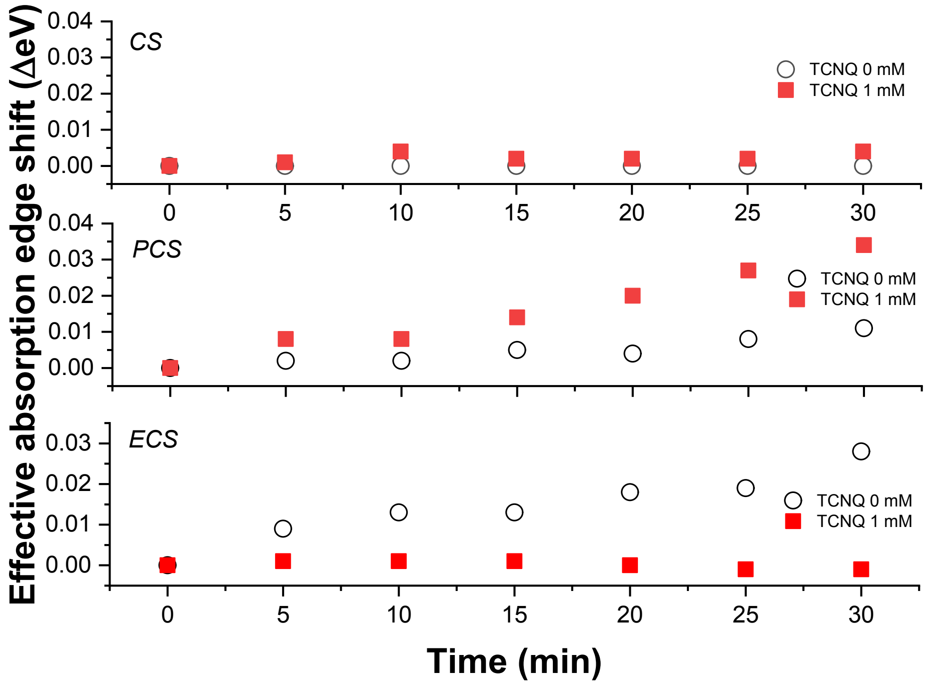

Figure S4. Effective absorption edge changes of $CsPbBr_3$ films under different stability testing condition. Details of each type of measurement can be found in **Table S2**.

Table S3. Summary of absorbance and photocurrents of $CsPbBr_3$ electrode during PEC measurement.

Condition	Abs @ 510 nm, 0 min	Abs (average)	current @1800s (μA cm^{-2})
TCNQ 0 mM	0.71	0.78 ± 0.09	-2.97
	0.71		-3.37
	0.90		-1.62
TCNQ 0.1 mM	0.71	0.65 ± 0.04	-26.5
	0.61		-20.8
	0.63		-18.8
TCNQ 1 mM	0.89	0.68 ± 0.18	-68.0
	0.70		-59.3
	0.46		-54.6

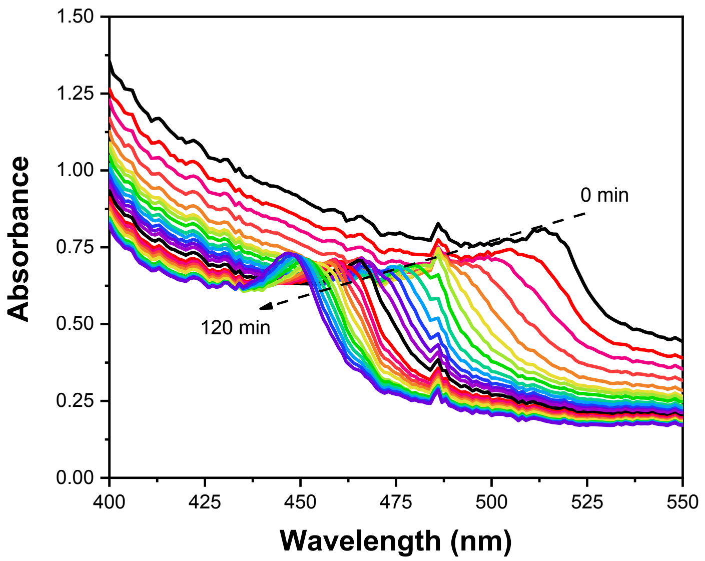

Figure S5. In-situ UV-vis absorption spectra of $CsPbBr_3$ films during PEC operation without TCNQ. During these measurements -0.2 V vs. Ag/AgCl was applied under spotlight irradiation (40 mW cm^{-2}) in 0.1 M Bu_4NPF_6 DCM electrolyte.

Determination of effective absorption edge

Briefly, as the first step the collected absorbance spectra was converted using the Tauc method. During this process we plotted to the $(\alpha h\upsilon)^n$ in the function of photon energy, where α is the determined absorption coefficient and the $h\upsilon$ is the photon energy. For a direct allowed transition (as in the case of $CsPbBr_3$) n=2 was used. On the obtained Tauc-plots (**Figure S6**) we determined the bandgap energy by linear fitting of the steep region of the curves (also performing correction for the non-characteristic absorption where necessary). This analysis is sufficient to determine the effective absorption edge in the case of low (or no) TCNQ concentrations. In the case of 1 mM TCNQ, the parasitic absorbance of the TCNQ was subtracted from all the spectra before performing the Tauc-analysis. For this we used the spectra recorded at the end of the halide-exchange reaction (60 minutes).

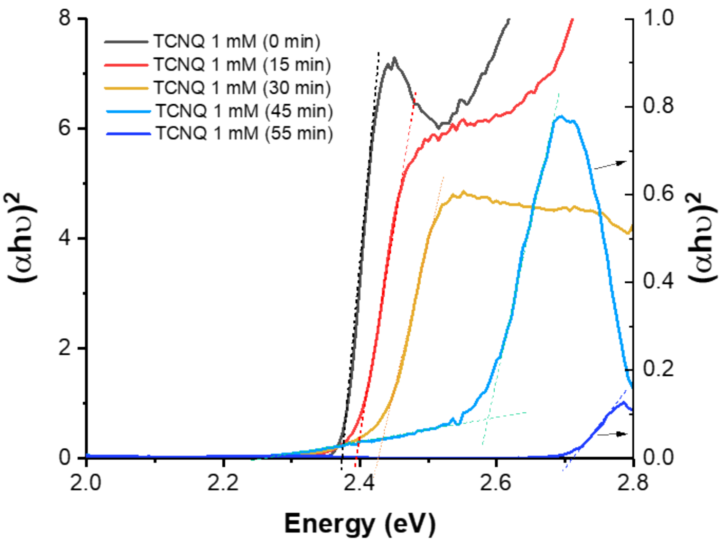

Figure S6. Estimation of effective absorption edge from Tauc plot for a $CsPbBr_3$ film in 1 mM TCNQ containing 0.1 M Bu_4NPF_6 DCM electrolyte at different stages of the PEC operation with an applied bias of -0.2 V vs. Ag/AgCl under spotlight irradiation (40 $mWcm^{-2}$).

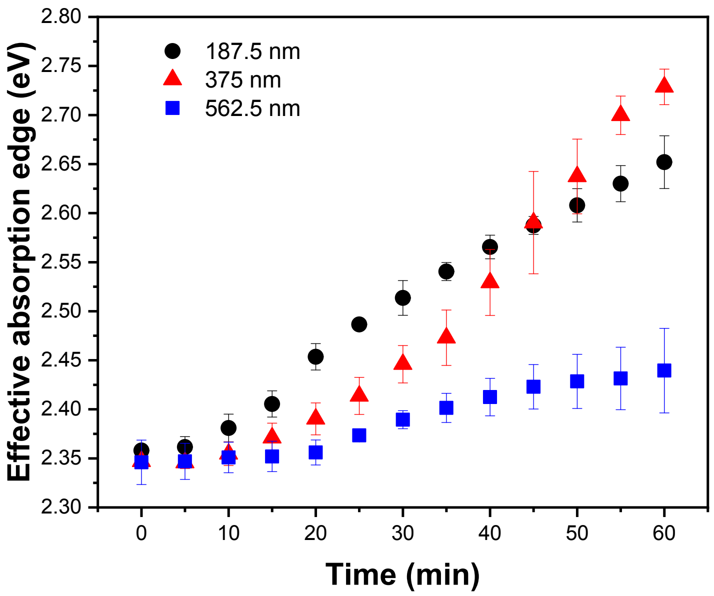

Figure S7. Time evolution of the effective absorption edge of $CsPbBr_3$ electrode in 0.1 mM TCNQ containing 0.1 M Bu_4NPF_6 DCM electrolyte during PEC operation with an applied bias of -0.2 V vs. Ag/AgCl under spotlight irradiation (40 $mWcm^{-2}$) with different film thickness. Error bars represent the standard deviation of measurements on at least two different $CsPbBr_3$ films.

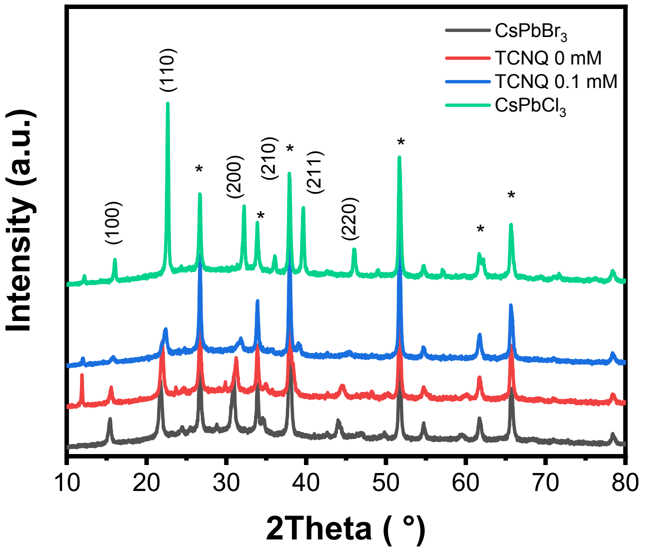

Figure S8. Full range X-ray diffraction patterns of pristine $CsPbBr_3$ and $CsPbCl_3$ films together with $CsPbBr_3$ photoelectrodes after 1 hour of PEC operation in 0.1 M Bu_4NPF_6/DCM media with and without TCNQ with an applied bias of –0.2 V vs. Ag/AgCl under spotlight (λ>400 nm) illumination with 40 mW cm^{-2}.

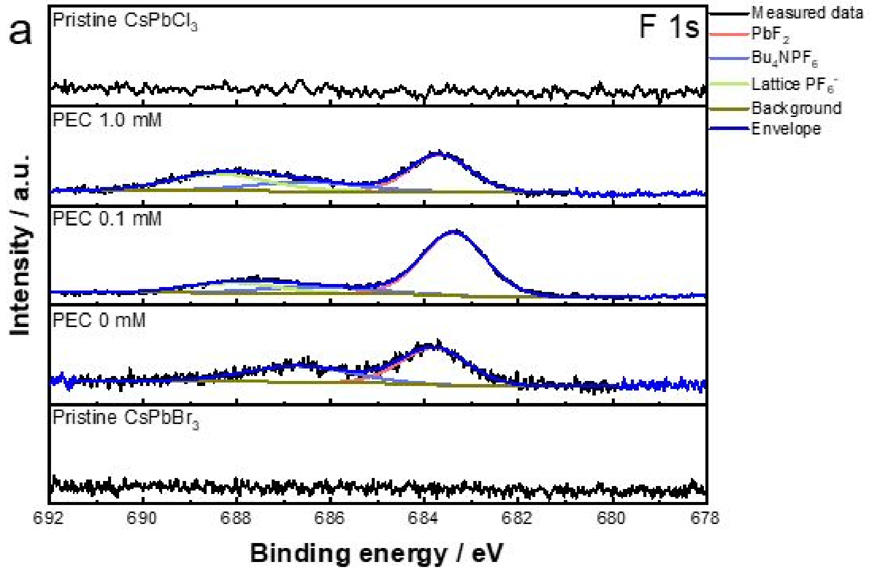

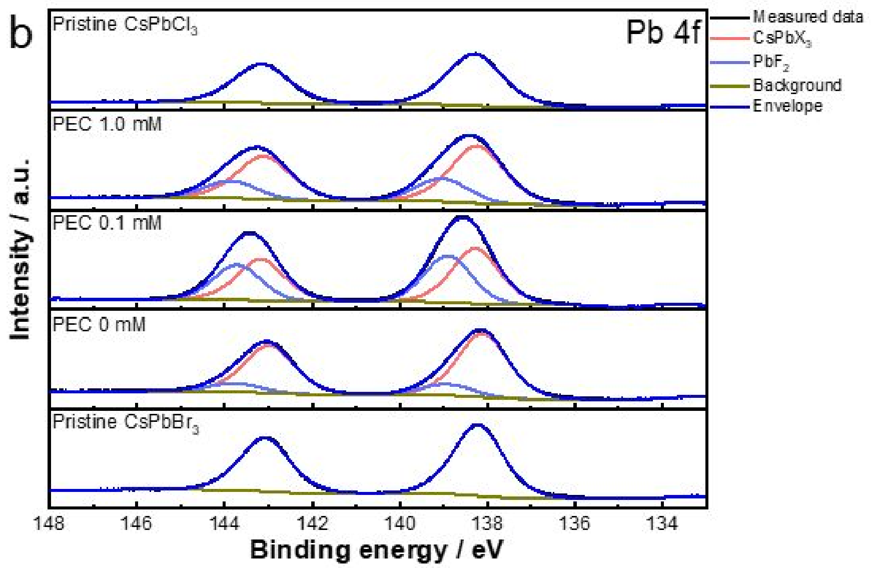
b
Pristine CsPbCl3
Pb 4f
Measured data
CsPbX3
PbF2
Background
Envelope
PEC 1.0 mM
PEC 0.1 mM
PEC 0 mM
Pristine CsPbBr3
Intensity / a.u.
148
146
144
142
140
138
136
134
Binding energy / eV

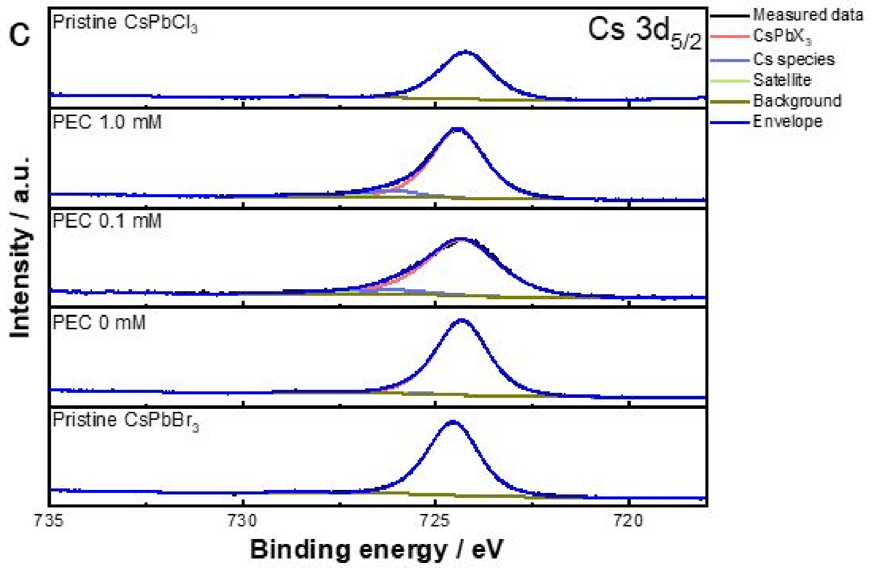
c
Pristine CsPbCl3
Cs 3d5/2
Measured data
CsPbX3
Cs species
Satellite
Background
Envelope
PEC 1.0 mM
PEC 0.1 mM
PEC 0 mM
Pristine CsPbBr3
Intensity / a.u.
735
730
725
720
Binding energy / eV

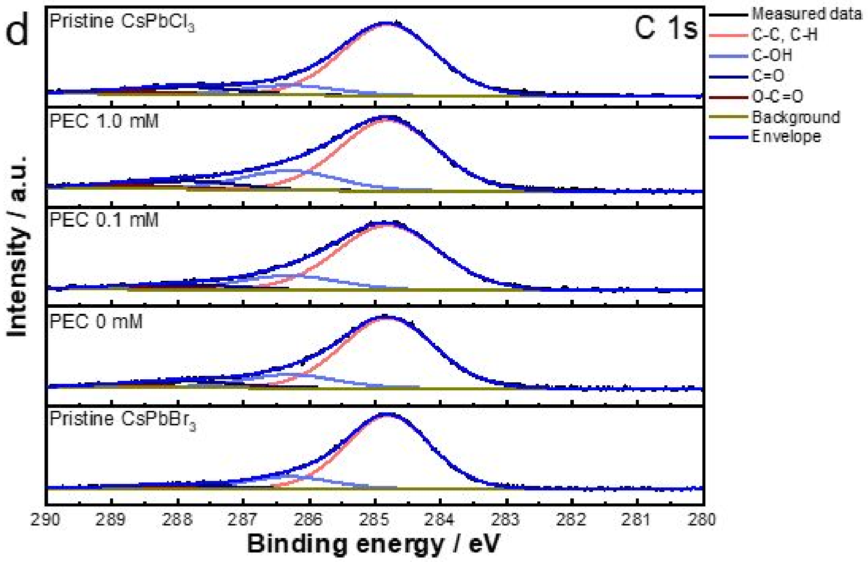

Figure S9. High resolution XPS spectra of the (a) F 1s, (b) Pb 4f, (c) Cs $3d_{5/2}$ and (d) C 1s core level region of the $CsPbBr_3$ electrodes after PEC measurements in 0.1 M Bu_4NPF_6 DCM electrolyte.

Table S4. Quantification of the surface composition of $CsPbX_3$ film after PEC operation at -0.2 V vs. Ag/AgCl for 1 hour in different media. Pristine $CsPbBr_3$ and $CsPbCl_3$ film were measured as a reference.

	**Pb content / at%**	**Cs content / at%**	**Br content / at%**	**Cl content / at%**	**F content / at%**
$CsPbBr_3$	21.3	20.1	58.6	-	-
$CsPbBr_3$ 0 mM TCNQ	21.3	19.8	43.6	7.8	7.5
$CsPbBr_3$ 0.1 mM TCNQ	29.0	8.04	14.1	17.3	31.6
$CsPbBr_3$ 1.0 mM TCNQ	21.6	18.2	4.0	41.4	14.8
$CsPbCl_3$	24.7	18.7	-	56.6	-

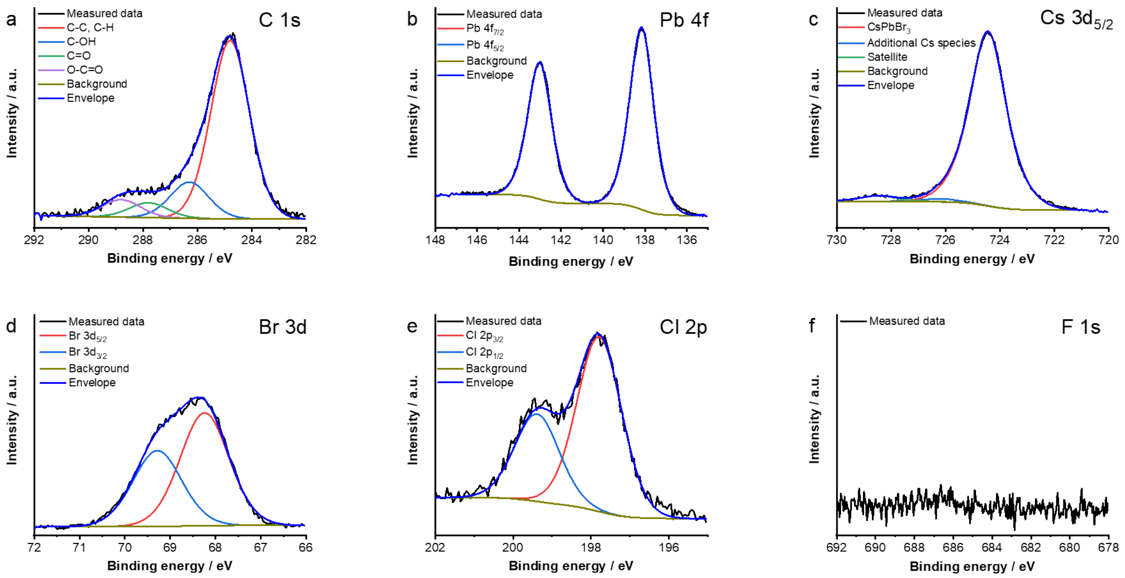

Figure S10. High resolution XPS spectra of the (a) C 1s, (b) Pb 4f, (c) Cs $3d_{5/2}$, (d) Br 3d, (e) Cl 2p and (f) F 1s core level region of the $CsPbBr_3$ electrodes after PEC measurements in 0.01 M Bu_4NBPh_4 DCM electrolyte.

Table S5. Quantification of the surface composition of $CsPbX_3$ film after PEC in 0.1 mM TCNQ containing 0.01M Bu_4NBPh_4 DCM media. A surface composition of $CsPbBr_{1.5}Cl_{1.5}$ was obtained.

	Pb content / at%	Cs content / at%	Br content / at%	Cl content / at%	F content / at%
$CsPbBr_3$ 0.1 mM TCNQ	20.0	20.5	29.7	29.8	-

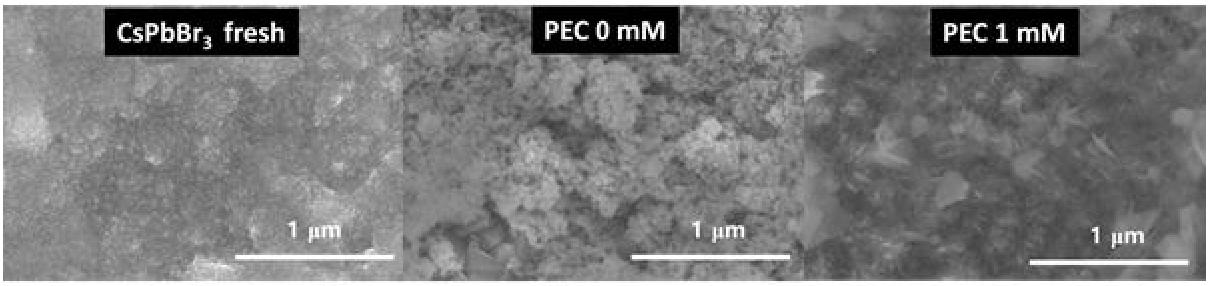

Figure S11. Top-down SEM images of $CsPbBr_3$ films before and after PEC operation.

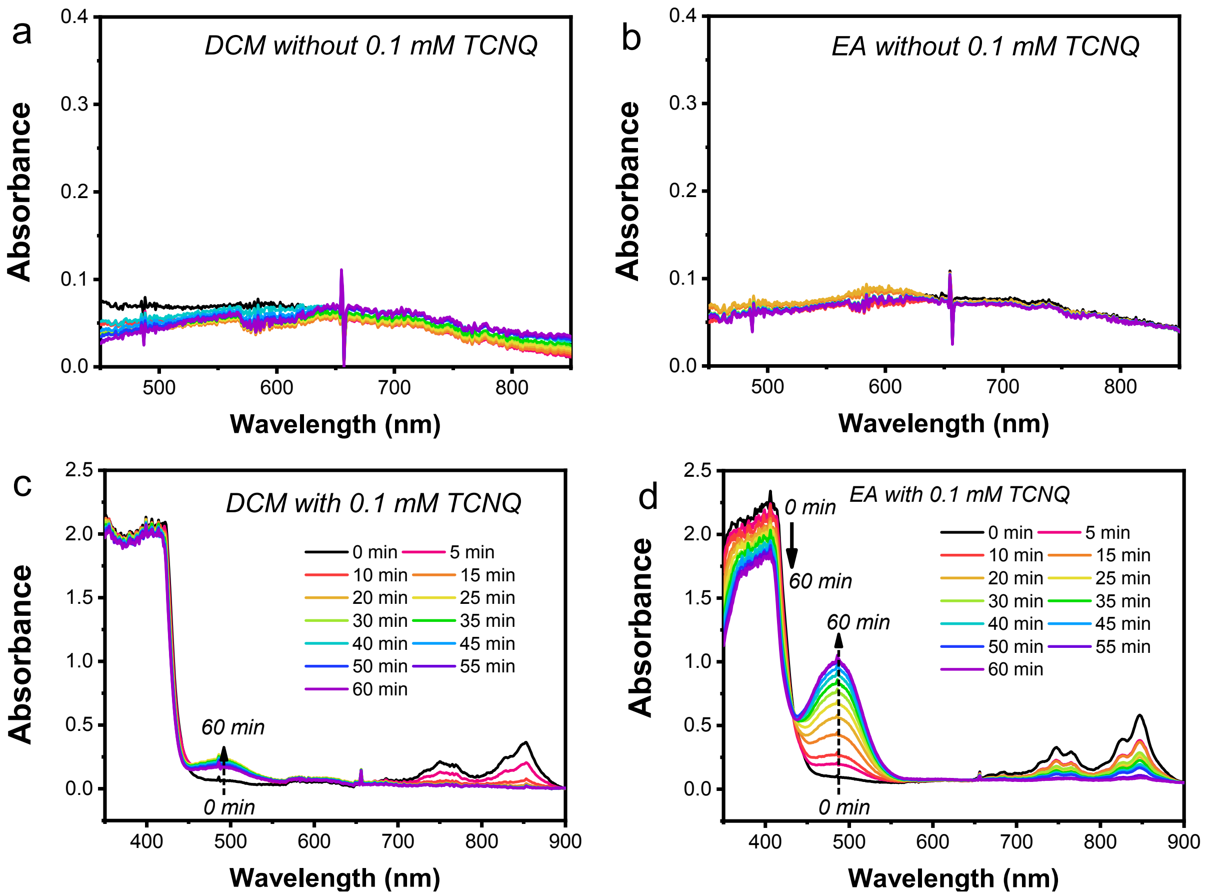

Figure S12. In-situ UV-vis absorption spectra of the electrolyte during PEC operation of a $CsPbBr_3$ electrode a, b: without and c, d: with added 0.1 mM TCNQ. All spectra -0.2 V vs. Ag/AgCl was applied under spot-light irradiation (40 mW cm^{-2}) with DCM (0.1 M Bu_4NPF_6) and EA (0.01 M Bu_4NPF_6) electrolyte.

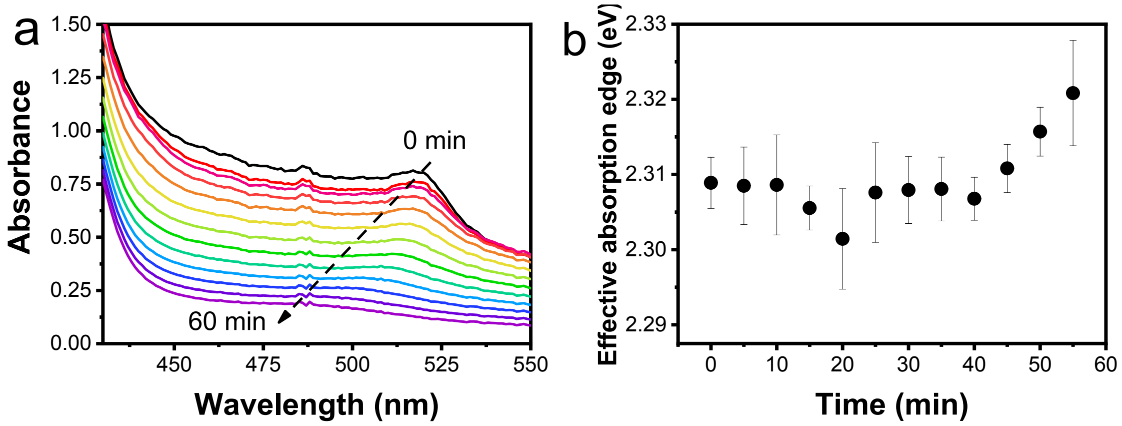

Figure S13. (a) In-situ UV-vis spectra and (b) the determined effective absorption edge of $CuI/CsPbBr_3$ electrodes recorded during PEC operation in 0.1 mM TCNQ containing 0.1 M Bu_4NPF_6 DCM solution with an applied bias of -0.2 V vs. Ag/AgCl under spotlight irradiation (40 $mWcm^{-2}$). Error bars represent the standard deviation of measurements on three different $CuI/CsPbBr_3$ films.

Extension to $MAPbI_3$ electrodes

To analyze whether this halide exchange is universal to LHPs or confined to $CsPbBr_3$, we performed similar experiments with $MAPbI_3$ photoelectrodes. In the presence of 1 mM TCNQ the linear sweep photovoltammograms showed a p-type behavior with a slightly higher maximum photocurrent value compared to $CsPbBr_3$ (**Figure S14a**). The large spikes on the photovoltammograms during illumination, however, signals enhanced surface recombination. Degradation was also confirmed by the large loss of photocurrent when performing a second scan in the same media. We performed spectroelectrochemical measurements under simultaneous illumination to track the previously observed halide exchange process (**Figure S14b** and **Figure S15**). Without TCNQ a stable optical absorbance is recorded for the films in the 0.1 M Bu_4NPF_6 DCM media (**Figure S15**). The stability of $MAPbI_3$ is slightly better compared to $CsPbBr_3$, which might be rationalized by the lattice mismatch between iodide and chloride, that poses a barrier for chloride incorporation into the layers. When 1 mM TCNQ was added, the simultaneous generation of the $TCNQ^-$ and a rapid decrease in the $MAPbI_3$

absorbance was witnessed. Interestingly in this case no clear bandgap shift is observed, ultimately resulting in PbI_2 (judging from the final effective absorption edge of 520 nm).

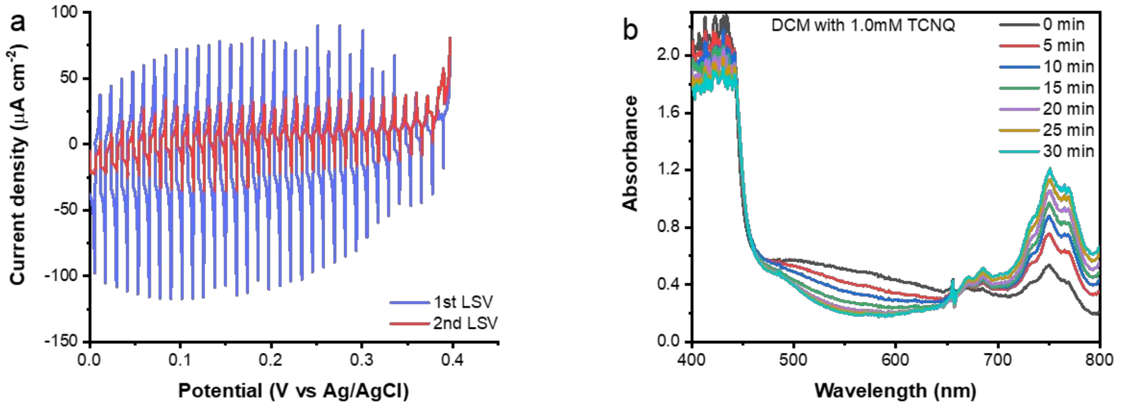

Figure S14. (a) Subsequent linear sweep photovoltammograms of FTO/$MAPbI_3$ electrodes with 1mM of TCNQ recorded with 2 mV s^{-1} sweep rate and a light chopping frequency of 0.3 Hz under AM1.5G solar light illumination with 100 mW cm^{-2} intensity (UV filtered). (b) In-situ UV-vis spectra of $MAPbI_3$ films during PEC operation at 0.0 V vs. Ag/AgCl applied potential under spotlight irradiation (40 mW cm^{-2}). Both measurements were carried out in 0.1 M Bu_4NPF_6 DCM electrolyte.

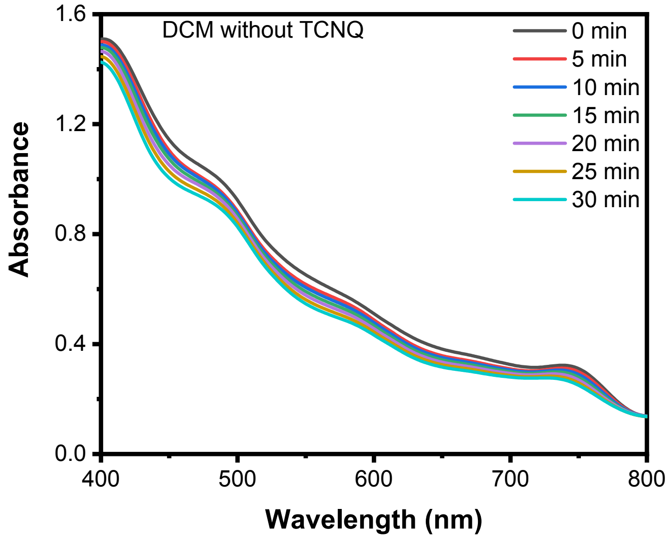

Figure S15. In-situ UV-vis spectra of $MAPbI_3$ films during PEC operation at 0.0 V vs. Ag/AgCl applied potential under spotlight irradiation (40 mW cm^{-2}) in 0.1 M Bu_4NPF_6 DCM electrolyte, without added TCNQ.

Table S6. Summary of the composition from different surface analysis techniques. Note that in the case of compositions determined from UV-vis, a 1:1 ratio of Pb:Cs was presumed.

	UV-vis [5]	EDX	XPS
$CsPbBr_3$	$CsPbBr_3$	$Cs_{1.2}PbBr_{3.60}$	$Cs_{0.9}PbBr_{2.8}$
$CsPbBr_3$ 0 mM TCNQ	$CsPbBr_{2.89}Cl_{0.14}$	$Cs_{1.1}PbBr_{3.26}Cl_{0.79}$	$Cs_{1.1}PbBr_{2.4}Cl_{0.4}$
$CsPbBr_3$ 0.1 mM TCNQ	$CsPbBr_{0.29}Cl_{2.71}$	$Cs_{1.1}PbBr_{0.92}Cl_{2.4}$	$Cs_{0.5}PbBr_{0.9}Cl_{1.1}$
$CsPbBr_3$ 1.0 mM TCNQ	$CsPbBr_{0.28}Cl_{2.72}$	$Cs_{1.6}PbBr_{0.44}Cl_{2.6}$	$Cs_{1.2}PbBr_{0.3}Cl_{2.6}$
$CsPbCl_3$	$CsPbCl_3$	$Cs_{0.81}PbCl_{2.4}$	$Cs_{0.8}PbCl_{2.3}$

Supplementary References

[1] A. Pan, B. He, X. Fan, Z. Liu, J.J. Urban, A.P. Alivisatos, L. He, Y. Liu, Insight into the Ligand-Mediated Synthesis of Colloidal $CsPbBr_3$ Perovskite Nanocrystals: The Role of Organic Acid, Base, and Cesium Precursors, *ACS Nano*, 10 (2016) 7943-7954.

[2] Á. Balog, G. F. Samu, P. V. Kamat & C. Janáky, Optoelectronic Properties of CuI Photoelectrodes. *The Journal of Physical Chemistry Letters*, 10.2 (2019), 259-264.

[3] D. Roy, G.F. Samu, M.K. Hossain, C. Janáky, K. Rajeshwar, On the measured optical bandgap values of inorganic oxide semiconductors for solar fuels generation, *Catalysis Today*, 300 (2018) 136-144.

[4] J.T. DuBose, P.V. Kamat, Surface Chemistry Matters. How Ligands Influence Excited State Interactions between $CsPbBr_3$ and Methyl Viologen, *The Journal of Physical Chemistry C*, 124 (2020) 12990-12998.

[5] U.-G. Jong, C.-J. Yu, J.-S. Ri, N.-H. Kim, G.-C. Ri, Influence of halide composition on the structural, electronic, and optical properties of mixed $CH_3NH_3Pb(I_{1-x}Br_x)_3$ perovskites calculated using the virtual crystal approximation method, *Physical Review B*, 94 (2016) 125139.
